# How continuing professional education interventions enhance the uptake of evidence-based practices among oncology nurses: a realist review protocol

**DOI:** 10.1136/bmjopen-2025-110800

**Published:** 2026-05-27

**Authors:** Billy Vinette, Guillaume Fontaine, Louis-Pierre Auger, Andrea Quaiattini, Aliki Thomas

**Affiliations:** 1 School of Physical and Occupational Therapy, McGill University, Faculty of Medicine and Health Sciences, Montreal, Quebec, Canada; 2 Centre for Interdisciplinary Research in Rehabilitation of Greater Montreal, Montreal, Quebec, Canada; 3 Institute of Health Sciences Education, McGill University, Montreal, Quebec, Canada; 4 Ingram School of Nursing, McGill University, Faculty of Medicine and Health Sciences, Montreal, Quebec, Canada; 5 Lady Davis Institute for Medical Research Centre for Clinical Epidemiology, Montreal, Quebec, Canada; 6 Centre for Nursing Research, Sir Mortimer B Davis Jewish General Hospital, Montreal, Quebec, Canada; 7 Methodological and Implementation Research Program, Ottawa Hospital Research Institute Centre for Implementation Research, Ottawa, Ontario, Canada; 8 Schulich Library of Physical Sciences, Life Sciences, and Engineering, McGill University, Montreal, Quebec, Canada

**Keywords:** ONCOLOGY, Review, Nurses, MEDICAL EDUCATION & TRAINING, Implementation Science, Education, Medical

## Abstract

**Abstract:**

**Introduction:**

Oncology nurses are pivotal to delivering high-quality cancer care; yet, the uptake of evidence-based practices (EBPs) remains inconsistent. Though continuing professional education is widely used to support EBP uptake, persistent gaps remain, likely driven by the intricate and interrelated mechanisms that unfold differently across individual, organisational and system contexts.

**Objective:**

To understand how, why, for whom and under what conditions continuing professional education interventions enhance (or fail to enhance) the uptake of EBPs among oncology nurses.

**Methods and analysis:**

This realist review will adhere to the Realist and Meta-narrative Evidence Syntheses: Evolving Standards methodological standards. The Theoretical Domains Framework will guide the exploration of potential mechanisms. 33 initial programme theories, developed from the Theoretical Domains Framework, prior reviews, expert input and consultations with interested parties working in oncology (eg, oncology nurses, managers), will be refined through systematic searches (CINAHL, MEDLINE, EMBASE, PsycINFO and Google Scholar). These initial programme theories represent hypothesised Context-Mechanism-Outcomes Configurations that may influence the uptake of EBPs among oncology nurses. Eligible peer-reviewed and grey literature from high-income countries in English or French will be screened in duplicate. Data will be coded deductively and inductively in MaxQDA and synthesised into Context-Mechanism-Outcome Configurations. These configurations will be reviewed in collaboration with interested parties.

**Ethics and dissemination:**

Findings will be disseminated through open-access, peer-reviewed publications and presentations at national and international conferences. Key stakeholders, including various professional associations (eg, the Canadian Association of Nurses in Oncology and the International Society of Nurses in Cancer Care), will be actively engaged to ensure the clinical relevance of the findings and to maximise their translation into nursing practice.

**PROSPERO registration number:**

CRD420251133710.

**STRENGTHS AND LIMITATIONS OF THIS STUDY**

- This realist review protocol follows the Realist and Meta-narrative Evidence Syntheses: Evolving Standards methodological standards, ensuring rigour, transparency and replicability.
- The Theoretical Domains Framework provides a comprehensive and theory-driven lens to identify and interpret mechanisms influencing the uptake of evidence-based practices in oncology nursing.
- The inclusion of a multidisciplinary Expert Advisory Committee such as oncology nurses, managers, researchers and professional associations representative strengthens the review’s practical relevance and interested parties’ engagement.
- Use of a multistage appraisal process (relevance, richness, rigour) and the TAPUPASM framework enhances methodological transparency and strengthens the trustworthiness of the evidence synthesis.
- The geographic and language restriction to English and French primary studies from high-income countries, may limit the transferability of the findings to low- and middle-income healthcare settings.

## Introduction

 Patients diagnosed with cancer are supported by a broad range of healthcare professionals (HCPs) across every stage of their journey, from diagnosis and treatment to survivorship to end-of-life care.^1^,^3^ Nurses are central to this continuum, providing direct care (eg, administering antineoplastic agents), coordinating complex care trajectories and promoting patient well-being through self-management education and psychosocial support.^4^,^8^

Given the complexity of oncology nursing care (eg, managing complications and infections, safely handling antineoplastic drugs and providing survivorship care for patients and those nearing the end of life), the adoption of evidence-based practices (EBPs) is crucial. While several interventions have been shown to increase EBP use among nurses,^49^,^11^ significant gaps persist.^12 13^ Numerous studies point to a constellation of barriers, spanning individual factors (eg, lack of confidence, skills, attitudes) and organisational or systemic barriers (eg, insufficient leadership support, resource availability).^14^,^18^ In oncology, nurses encounter additional challenges, including deficits in EBP knowledge (eg, antineoplastic drugs handling standards), difficulties integrating evidence into practice change and limited engagement in evidence appraisal or sustaining an EBP culture.^9 10 19^

To address these barriers, a range of implementation strategies, such as those outlined in the Expert Recommendations for Implementing Change (ERIC) taxonomy,^20^ have been developed to drive EBP uptake and shift professional practice, including in nursing.^1221^,^23^ A recent meta-analysis of 204 studies confirmed that these strategies can effectively improve nursing practices, although their success often depends on contextual conditions.^24^ Continuing professional education (CPE) interventions such as group-based training or online modules are a critical implementation strategy commonly employed across healthcare settings.^25^,^27^ We have evidence that these interventions can improve different outcomes such as knowledge, skills and social norms.^24^

While some CPE interventions are effective, implementation strategies do not operate in a vacuum. A growing body of evidence underscores that strategies are shaped by the settings in which they are applied.^21 28 29^ Enabling and hindering contextual factors play a critical role in how HCPs, including nurses, engage with EBPs.^2830^,^32^ These factors, which are both dynamic and evolving, are adding complexity to the implementation process.^33^ Understanding these shifting contexts is crucial for guiding the choice, design and implementation of EBP strategies to enhance the success and sustainability of EBPs in nursing practice. Scholars increasingly advocate for more nuanced, context-sensitive approaches such as realist reviews to better understand how and why implementation strategies work (or fail).^12 21 29 33 34^ Realist methods are particularly suited to uncovering the mechanisms that drive change and how these mechanisms are activated-or not across diverse contexts, populations and points in time.^35^,^37^ Despite the widespread use of CPE to promote EBPs in oncology nursing, we still lack a clear understanding of how, why, for whom and under what conditions these interventions are effective.

### Aim

This review aims to explore the mechanisms and contextual factors that influence the success of CPE in supporting the uptake of EBP among oncology nurses.

### Methods

### Realist review methodology

A realist review adopts a non-linear, iterative approach, rooted in realist philosophy, to synthesise evidence from a wide range of sources, including primary studies, grey literature and policy documents.^38^,^42^ Its central aim is to understand how and why programmes (ie, interventions) work, for whom and under which conditions.^38^,^42^ To achieve this, realist reviews examine the causal links between contexts, mechanisms and outcomes.^39 41^

Context refers to the conditions that can trigger or hinder mechanisms,^38 40 43^ such as organisational support, physical settings, available resources or policies. Mechanisms are dynamic processes that explain why and how outcomes occur, or fail to occur.^42^ As context-sensitive elements, mechanisms can operate concurrently or interact with one another and produce both intended and unintended outcomes.^40 42 44 45^

Outcomes refer to the intended or unintended effects of an intervention resulting from the interactions between contextual factors and mechanisms.^38 40^ In this review, we conceptualise the uptake of EBPs as an intermediate outcome shaped by the activation (or not) of specific mechanisms within particular contexts. Outcomes may manifest across multiple levels, including:

- Patient-level outcomes, such as patient-reported experience measures and patient-reported outcome measures.
- Provider-level outcomes, such as clinical decision process^46^ or adherence to personal protective equipment use.^47^
- Implementation outcomes, as listed and defined by Proctor *et al*,^48^ including acceptability, adoption, appropriateness, feasibility, fidelity, implementation cost, penetration and sustainability.

The combination of contextual factors (C), mechanisms (M) and outcomes (O) is expressed through Context-Mechanism-Outcome Configurations (CMOCs). Consistent with realist logic, mechanisms are understood as the cognitive, emotional or motivational processes triggered or constrained by specific contextual conditions. CMOCs form the basis of a programme theory, the central output of a realist review, which explains why and describes how an intervention is expected to work, by unpacking the complex relationships between these key elements.^38 40 42 43^

### Designing the review

To achieve this, our review will follow the five iterative stages proposed by Pawson *et al*,^41^ namely: (1) define the scope and form the Expert Advisory Committee; (2) search for evidence; (3) appraise documents and extract data; (4) synthesise evidence and draw conclusions; and (5) disseminate, implement and evaluate.

**Figure 1:**
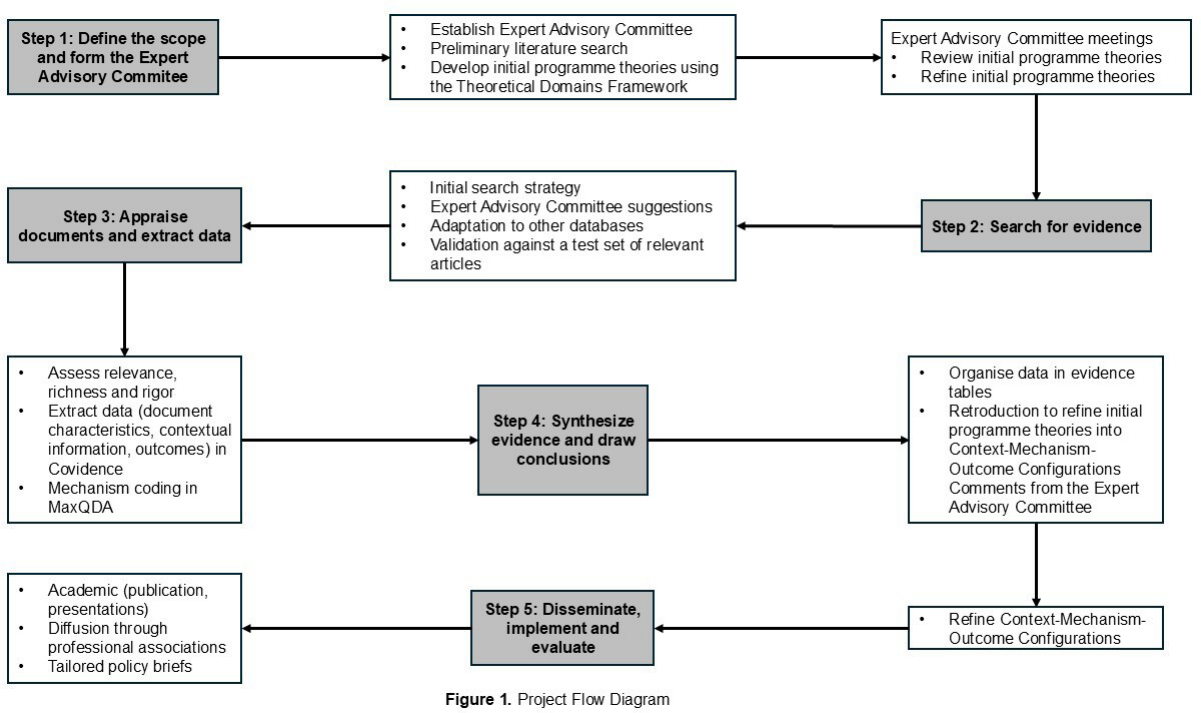
Project flow diagram.

This review will be conducted in accordance with the Realist and Meta-narrative Evidence Syntheses: Evolving Standards guidelines for realist syntheses.^39^ Additionally, the Preferred Reporting Items for Systematic Review and Meta-Analysis Protocols (PRISMA-P)^49^ is used to enhance the transparency, clarity and replicability of this protocol (see online supplemental file S1). The review protocol was registered at PROSPERO prior to undertaking the literature search (CRD420251133710). A detailed project flow diagram is presented in figure 1.

### Patient and public involvement

No patients or members of the public were involved in the design of the study, the definition of the research question or the selection of outcome measures. However, the findings of this review will be shared with patient advocacy groups during the dissemination phase to ensure that the resulting practice recommendations reflect patient priorities.

#### Step 1: define the scope and form the Expert Advisory Committee

##### Define the scope

Our multidisciplinary team, combining clinical expertise (BV and L-PA), methodological expertise (AT and GF) and an academic medical librarian (AQ), conducted a preliminary literature scan (Google Scholar, PubMed) to explore the current evidence on the adoption of EBPs among nurses. This initial search suggested that barriers are complex and can arise at multiple levels: individual, organisational and systemic.^50^ Moreover, the roles and responsibilities of nurses, as the primary target audience, can influence how implementation strategies unfold.^12^ It also identified several recent systematic reviews,^12 23 24 50 51^ which highlighted key questions and evidence gaps that shaped our realist review, including:

1. What contextual factors at the individual, organisational or systems level enhance or hinder the uptake of EBPs among oncology nurses following a CPE intervention?
2. What mechanisms are triggered within the context following a CPE intervention that enhance or hinder the uptake of EBPs among oncology nurses?
3. To what extent do these contexts and mechanisms vary across settings and specific evidence-based nursing practices?
4. What are the anticipated and unanticipated outcomes, both positive and negative, among oncology nurses associated with the uptake of EBPs following a CPE intervention?

##### Develop initial programme theories

As theory-driven methodologies, realist reviews draw on programme theories, which may take various forms, including existing theories, to unravel the complex interplay between contextual factors (eg, organisational support), mechanisms and outcomes that shape the success or failure of complex interventions.^38^,^4152^

In recent years, theories, models and frameworks (TMFs) from Implementation Science have been increasingly integrated into realist evaluations, notably to support the uptake of EBPs among HCPs.^3853^,^60^ TMFs are recognised to provide a coherent structure to organise and interpret complex data. They support the development of CMOCs and help identify implementation strategies likely to influence contextual factors and enhance practice change among HCPs.^53 57 61 62^ Leveraging TMFs and their underlying principles could offer a robust understanding for understanding how and why EBP uptake varies across settings.

For this review, we adopted the Theoretical Domains Framework (TDF), a widely used framework in Implementation Science that integrates key constructs from multiple behaviour change theories. The TDF was selected based on attributes identified by Fontaine *et al*,^60^ including clarity of constructs, parsimony, evidence-based, practicality, relevance to health or healthcare, consistency with multidisciplinary and adaptability. Originally developed to identify and explain factors influencing the uptake of interventions among HCPs,^63^ the TDF encompasses 14 domains spanning knowledge, skills, cognitive, emotional, social and contextual influences on behaviour. While not a formal theory typically applied in realist evaluations, the TDF offers a robust conceptual scaffold for examining multilevel influences. Its comprehensive structure and clearly defined domains enhance analytical consistency and support theory-informed interpretation of findings that may be particularly relevant in oncology nursing settings.^63 64^

We developed 33 initial programme theories (IPTs) (see online supplemental file S2) using a deductive approach informed by the TDF,^63^ insights from a prior realist review on HCPs delivering self-management support to patients diagnosed with cancer,^38^ and the experiential knowledge of our team, including an oncology nurse (BV) and HCPs from two additional professions (L-PA, GF and AT). The IPTs represent hypothesised context-mechanism configurations that may influence the uptake of EBPs among oncology nurses. These will be progressively refined into full CMOCs during subsequent stages of the review.

Members of the Expert Advisory Committee will be invited to prioritise the most relevant IPTs, those deemed most influential for EBP uptake in oncology nursing, during dedicated consultation meetings.

##### Form the Expert Advisory Committee

As per realist review methodology,^40 41^ an Expert Advisory Committee will be formed to provide iterative input and ensure the review remains grounded in practice. The committee will include a diverse range of interested parties, such as: registered nurses working in oncology (eg, radio-oncology, haematopoietic units or outpatient clinics), nurse navigators, clinical nurse specialists, nurse practitioners, nurse managers, a nurse affiliated with a provincial cancer agency (Quebec Cancer Program), representatives from both national and provincial oncology professional nursing associations and academic researchers (BV, GF, L-PA and AT).

Committee members will be intentionally recruited through the research team’s professional networks and invited to engage in: (1) providing feedback on the research questions and reviewing the protocol; (2) contributing additional search terms and suggesting key literature; (3) reviewing and refining IPTs; and (4) commenting on the interpretation of findings and CMOCs.

To gather insights and practical perspectives that can inform our understanding of the topic, multiple meetings will be held throughout the project with committee members. For members unable to attend in real time, input will be sought asynchronously via email (see online supplemental file S3). This engagement strategy will support the iterative and stakeholder-driven nature of the realist review.

### Search for evidence

The keywords and Medical Subject Headings, and overall search strategy (see table 1 and online supplemental file S4) were developed with an academic medical librarian (AQ) to ensure methodological rigour and relevance. It targeted three concepts: (1) nurses (eg, nursing); (2) oncology (eg, patients with cancer, leukaemia, lymphoma); and (3) continuing professional education (eg, in-service training). The initial strategy was designed for MEDLINE (Ovid) and will be subsequently adapted for Cumulative Index to Nursing and Allied Health Literature (CINAHL; EBSCOhost), EMBASE (Ovid) and PsycINFO (Ovid). Google Scholar will also be searched systematically using the keywords ‘Nurses/Nursing’, ‘Oncology’ and ‘Continuing Education’, with the first 250 results screened for potentially relevant studies. We will also manually search the repositories of major oncology nursing organisations (eg, Oncology Nursing Society (ONC), International Society of Nurses in Cancer Care (ISNCC), Canadian Association of Nurses in Oncology (CANO), Cancer Nurses Society of Australia) to retrieve relevant grey literature, reports and unpublished studies that meet our inclusion criteria.

**Table 1:** Keywords and MeSH used in MEDLINE

Concepts	Keywords and MeSH
Nurses	(MH “Nursing”) (MH “Nurses”) (MH “Nursing Staff”) (MH “Oncology Nursing”) Nurse, Nursing.
Oncology	(MH “Neoplasms”) Adenolymphoma, Adenoma, Adenomyoepithelioma, Adenomyoma, Adenosarcoma, Angiofibroma, Angiomyolipoma, Astrocytoma, Cancer, Carcinoma, Carcinosarcoma, Carcinoid, Chondroma, Chondroblastoma, Chondrosarcoma, Choriocarcinoma, Craniopharyngioma, Cystadenocarcinoma, Cystadenoma, Dermatofibrosarcoma, Ependymoma, Fibroma, Fibrosarcoma, Ganglioma, Germinoma, Glioblastoma, Glioma, Hemangioma, Hemangioendothelioma, Hemangiosarcoma, Hepatoblastoma, Hodgkin, Insulinoma, Leiomyoma, Leiomyomatos, Leiomyosarcoma, Leukemia, Lesion, Lipoma, Liposarcoma, Lymphangioleiomyomatos, Lymphoma, Mastocytos, Medulloblastoma, Melanoma, Meningioma, Mesenchymoma, Mesothelioma, Metastasis, Metastatic, Myeloma, Myoma, Myosarcoma, Myxoma, Neoplasm, Neurilemmoma, Neuroblastoma, Oncologic, Oncology, Oligodendroglioma, Oseoblastoma, Osteochondroma, Osteoma, Osteosarcoma, Paraganglioma, Pinealoma, Plasmacytoma, Retinoblastoma, Sarcoma, Teratoma, Thymoma, Tumor, Tumour.
Continuing professional education	(MH Education, Continuing”) (MH “Inservice Training”) (MH “Staff Development”) (MH “Evidence-Based Practice”) (MH “Evidence-Based Medicine”) (MH “Evidence-Based Nursing”) Continuing Development, Continuing Education, CPE, CPD, EBM, EBN, EBP, Employee Development, Employee Education, Evidence-Based, Evidence-Informed, In service Training, Inservice Training, Staff Development, Staff Education, Worker Development, Worker Education.

MeSH, Medical Subject Headings.

To enhance comprehensiveness, we will add forward and backward citation tracking of included studies. The search strategy was validated against a test set of relevant articles to ensure adequate coverage.^910 46 47 65^,^68^ We anticipate making iterative refinements throughout the review process; any adjustments and their justifications will be documented in the final paper.

### Appraise documents and extract data

#### Document appraisal

Recognising the diverse sources that can inform theory development,^39 40^ we will include both academic and grey literature (eg, reports, conference proceedings), regardless of methodological design, provided they are primary studies. Knowledge syntheses will be excluded. We will restrict our selection to documents published in English or French. To ensure contextual relevance, particularly regarding socioeconomic (eg, income levels) and health system characteristics (eg, organisation of care delivery), we will limit inclusion to studies conducted in high-income countries, as classified by the World Bank.^69^

To guide document selection, we will apply established realist review criteria: relevance (contribution to theory development), richness (depth of explanation) and rigour (trustworthiness and methodological quality).^39^,^4170^ Given that this process is often inconsistently reported and operationalised,^70^ we will employ a multistage selection process that will be refined iteratively throughout the review (detailed below). Stage 1 will assess relevance, Stage 2 will evaluate richness and Stage 3 will address rigour. Title and abstract screening, followed by full-text review, will be conducted independently by two reviewers (BV and L-PA). All screening stages will be pilot-tested to ensure consistency in the application of the criteria. Discrepancies will be resolved through discussion with a third reviewer (AT).

#### Stage 1: relevance

Documents will be screened for relevance using predefined quality appraisal criteria within Covidence (Veritas Health Innovation, Australia) (see table 2). Eligible documents will address the implementation, uptake or sustainability of EBPs in the context of CPE interventions targeting oncology nurses. To identify the implementation strategies described, we will use the ERIC Taxonomy,^20^ which outlines 73 discrete strategies, including audit and feedback, facilitation and engagement of local opinion leaders.

**Table 2:** Quality appraisal criteria

Step 1: relevance	
High relevance	Describe oncology nurses’ views, experiences and needs with the implementation, uptake or sustainability of EBPs in the context of CPE interventions. Describe context, facilitators or barriers associated with the implementation, uptake or sustainability of EBPs among oncology nurses in the context of CPE interventions. Describe studies on the perspectives of patients diagnosed with cancer, their caregivers or non-nursing healthcare professionals on oncology nurses’ uptake of EBPs in the context of CPE interventions. Describe implementation strategies used to support oncology nurses’ uptake of EBPs in the context of CPE interventions (eg, local needs assessment, champions, reminders). Describe implementation outcomes related to oncology nurses’ uptake of EBPs in the context of CPE interventions (eg, adoption, sustainability). Describe oncology nurses’ behavioural outcomes in the context of CPE interventions that included an implementation strategy to increase uptake of EBPs (eg, knowledge, skills, decision-making). Describe oncology nurses’ uptake of EBPs either in general or in relation to a specific clinical practice (eg, dressing change, infection control) in the context of CPE interventions.
Moderate relevance	Same as high relevance, but oncology nurses represent less than 50% of the total healthcare professional sample. Same as high relevance, but patients diagnosed with cancer represent less than 50% of the total sample. Describe oncology nurses’ uptake of EBPs in the context of CPE interventions, where implementation strategies are present but insufficiently described.
Low relevance	Describe the implementation, uptake or sustainability of EBPs by healthcare professionals working in oncology in the context of CPE interventions, without a specific focus on nurses.
No relevance	Describe stand-alone CPE interventions with oncology nurses without any implementation strategies to support the uptake of EBPs (eg, reminders, audit).
Step 2: conceptual richness	
Conceptual rich	Explicitly reference TMFs underpinning the CPE intervention or related to oncology nurses’ uptake of EBPs. Offer rich description of implementation context, and/or mechanisms influencing oncology nurses’ uptake of EBPs. Contributes substantially to developing or refining CMOCs.
Conceptually thick	Describes context, or elements related to oncology nurses’ uptake of EBPs, but does not reference TMFs explicitly. Offers moderate explanatory value for CMOCs development.
Conceptually thin	Offers minimal or superficial description of implementation context, or uptake mechanisms. Lacks clear utility in contributing to theory building or CMOCs.
Step 3: contextual thickness	
Contextual rich	Provides detailed descriptions of the intervention’s implementation (eg, components, delivery process) and the surrounding context (eg, setting, organisational conditions). Readers can easily assess the intervention’s transferability to other contexts.
Contextual thick	Offers some description of either the intervention’s implementation or its context, but not both. Readers may find it challenging, though still possible, to assess transferability.
Contextual thin	Provides minimal or vague information about the intervention or its context, limiting the ability to assess transferability.
Step 4: rigour	
Transparency	Is the knowledge generation process transparently reported and sufficiently detailed to ensure clarity and understanding?
Accessibility	Does it address the needs of the researchers or the authors?
Propriety	Is the research conducted in accordance with ethical and legal standards?
Utility	Is the research appropriate and does it offer insights to practical questions?
Purposivity	Are the methods appropriately justified and aligned with the aims and objectives?
Accuracy	Are the claims grounded in relevant and appropriate information?
Specificity	Does the research align with methodological standards of quality?
Modified objectivity	Does the research use diverse sources of evidence to support the most credible conclusions?
Step 5: screening outcome	
Include	Meets at least moderate relevance and demonstrates conceptual and contextual richness or thickness. It shows sufficient rigour across TAPUPASM dimensions. It contributes meaningfully to the development or refinement of CMOCs.
Maybe	Meets moderate or low relevance, and provides some conceptual or contextual detail, but may have uncertainties regarding rigour. It may still offer partial insights or be useful for the refinement of CMOCs.
Exclude	Is of no relevance or provides conceptually and contextually thin data. It lacks rigour and does not support theory building or CMOCs development.

CMOC, Context-Mechanism-Outcome Configurations; CPE, continuing professional education; EBP, evidence-based practices; TMF, theories, models or frameworks.

#### Stage 2: richness

Each document will be assessed for richness across two dimensions: conceptual richness, referring to the extent to which data illuminate underlying mechanisms and contextual thickness, which reflects the granularity of contextual detail necessary to assess transferability.^70 71^ Conceptual richness will be categorised as rich, thick or thin, based on criteria adapted from Calderón-Larrañaga *et al*,^72^ while contextual thickness will be evaluated using the same classification (ie, rich, thick or thin).

#### Stage 3: rigour

Rigour will be assessed using the TAPUPASM framework,^73^ an adaptation of the TAPUPAS^74^ developed for critical realist research. This framework assesses the trustworthiness of documents across eight dimensions: transparency, accessibility, propriety, utility, purposivity, accuracy, specificity and modified objectivity.

Transparency refers to the extent to which the knowledge generation process is clearly documented, explicitly reported and open to scrutiny.^37 73^ Accessibility assesses whether the research is presented in a manner that meets the informational needs of intended users, such as researchers or interested parties.^37 73^ Propriety evaluates whether the research adheres to established ethical and legal standards.^37 73^ Utility concerns the extent to which the research provides actionable insights relevant to practice or policy.^37 73^ Purposivity examines whether the methods are appropriately justified and aligned with the aims and objectives.^37 73^ Accuracy refers to how well claims are grounded in relevant and appropriate information.^37 73^ Specificity assesses whether the research aligns with recognised methodological standards of quality.^37 73^ Modified objectivity considers the degree to which multiple and diverse sources of evidence are used to support the most credible conclusions.^73^

The rigour of each document will be classified as good, fair or poor. These assessments will then inform inclusion decisions, which will be documented in a Quality Appraisal Form (see table 3).

**Table 3:** Quality Appraisal Form

Authors (year)	Country	Type of paper	Study design	Relevance	Richness	Thickness	Rigour

#### Data extraction

Data will be extracted in Covidence (Veritas Health Innovation, Australia) using a data extraction form developed from the IPTs and informed by previous realist reviews on related topics (see table 4 and online supplemental file S5). This form will be refined iteratively during the extraction process, as needed.

**Table 4:** Data extraction form

Document information		Context						Outcomes		
Authors (year)	Document type	Country	Health system level	Design	Population	Intervention	Implementation strategy	Implementation outcomes	Provider-level outcomes	Patient-level outcomes

During the first phase, we will extract key characteristics of each document (eg, authors, publication year, document type), contextual information (eg, country, level of the health system, study design, target population, description of the intervention and implementation strategies employed), as well as reported outcomes (eg, implementation, provider-level and patient-level outcomes). Implementation strategies will be categorised using the ERIC taxonomy.^20^ Data extraction will be pilot-tested on a sample of 10 documents by two independent reviewers (BV and L-PA). On achieving satisfactory inter-rater consistency, 10% of the remaining documents will be independently extracted by both reviewers. After this, BV will proceed with extracting the remaining data. Any discrepancies will be resolved through discussion or, if necessary, by consulting a third reviewer with expertise in realist reviews (AT).

In the second phase, full-text documents will be imported into MaxQDA (VERBI Software) for in-depth coding of underlying mechanisms. Mechanisms will be coded both deductively, guided by the TDF domains^63^ and inductively. This phase will also be pilot tested on a sample of 10 documents by two independent reviewers (BV and L-PA). Following the establishment of a high level of inter-coder consistency, BV will proceed with coding the remaining documents. At the midpoint of the coding process, once approximately 50% of the documents have been completed, BV will consult with a realist review expert (AT) to ensure conceptual coherence and consistency in the identification and interpretation of mechanisms across the dataset. A structured coding manual (table 5) will be used to promote consistency and theoretical coherence across data sources.

**Table 5:** Coding manual

Constructs	Definitions	Code
Knowledge	An awareness of the existence of something	KNOW
Skills	An ability or proficiency acquired through practice	SKILLS
Social/professional role and identity	A coherent set of behaviours and displayed personal qualities of an individual in a social or work setting	ROLE&IDEN
Beliefs about capabilities	Acceptance of the truth, reality or validity about an ability, talent or facility that a person can put to constructive use	CAPA
Optimism	The confidence that things will happen for the best or that desired goals will be attained	OPT
Beliefs about consequences	Acceptance of the truth, reality or validity about outcomes of a behaviour in a given situation	CONSE
Reinforcement	Increasing the probability of a response by arranging a dependent relationship, or contingency, between the response and a given stimulus	REINF
Intentions	A conscious decision to perform a behaviour or a resolve to act in a certain way	INT
Goals	Mental representations of outcomes or end states that an individual wants to achieve	GOALS
Memory, attention and decision processes	The ability to retain information, focus selectively on aspects of the environment and choose between two or more alternatives	ME&AT&DE
Environmental context and resources	Any circumstance of a person’s situation or environment that discourages or encourages the development of skills and abilities, independence, social competence and adaptive behaviour	CON&RES
Social influences	Those interpersonal processes that can cause individuals to change their thoughts, feelings or behaviours	SOCIAL
Emotion	A complex reaction pattern, involving experiential, behavioural and physiological elements, by which the individual attempts to deal with a personally significant matter or event	EMOT
Behavioural regulation	Anything aimed at managing or changing objectively observed or measured actions	REGUL

Extracted data, including coding outputs and data extraction forms, will be securely stored and systematically versioned using a shared, access-restricted OneDrive folder (Microsoft Corporation) that is accessible to the review team.

### Synthesise evidence and draw conclusions

The analysis will follow an iterative, two-stage process of theoretical refinement. First, the data extracted via Covidence (Veritas Health Innovation, Australia) regarding contexts and outcomes will be used to test the viability of the IPTs. This step involves assessing the coherence of the IPTs against the evidence, identifying recurring patterns and highlighting gaps or inconsistencies where the data does not align with the initial theories.

Once the IPTs are stabilised, a second phase of in-depth coding will be conducted in MAXQDA (VERBI Software) to identify the latent mechanisms. Through a retroductive approach combining inductive and deductive reasoning, we will triangulate the coded mechanisms with the previously extracted contexts and outcomes. This synthesis will transform the IPTs into refined CMOCs, articulated as: ‘if [context]… then [outcome]… because [mechanism]’ propositions. BV and AT will engage in critical dialogue to challenge and strengthen interpretations. The resulting CMOCs will be displayed in tables and supported by selected quotes from the included documents to illustrate the underlying reasoning. We will also consider developing worked examples to enhance the presentation of results as suggested by Wong *et al*.^39^ The resulting CMOCs and examples will be reviewed with the research team and the Expert Advisory Committee to ensure both conceptual clarity and practical relevance.

### Disseminate, implement and evaluate

Findings from this realist review will be disseminated through a multifaceted strategy designed to reach both academic and professional audiences, including peer-reviewed publications and conference presentations. In parallel, we will engage key interested parties—including professional associations such as the CANO—Association canadienne des infirmières en oncologie (ACIO), the ISNCC and the ONS—through targeted discussions, tailored briefs and collaborative dissemination efforts. To optimise reach and relevance, we will develop audience-specific briefs and dissemination materials adapted to the contexts of both the Canadian and Quebec healthcare systems. These materials will be shared with key stakeholders in oncology nursing leadership and policy—including the Quebec Cancer Program and the Office of the Chief Nursing Officer of Canada.

We will share the findings of this realist review using a range of strategies tailored to both academic and frontline practice audiences. This includes peer-reviewed articles and conference presentations, as well as more accessible formats such as short, action-oriented briefs. We will work closely with key partners, including CANO-ACIO, ISNCC and ONS, to co-develop and circulate these materials. To ensure practical relevance, we will tailor the content to reflect the realities of both the Canadian and Quebec healthcare systems. Key stakeholders, such as the Quebec Cancer Program and the Office of the Chief Nursing Officer of Canada, will receive summaries focused on how the findings can inform oncology nursing practice and policy.

#### Timeline of the review

At the time of manuscript submission (September 2025), the search strategy had been completed in MEDLINE (Ovid) and will be adapted for the other databases, with searches scheduled to begin shortly thereafter. Title and abstract screening, along with full-text screening, is anticipated to be completed by October 2025. Data extraction and coding are expected to be finalised by December 2025, followed by evidence synthesis and refinement of CMOCs by February 2026. The results and manuscript are expected to be submitted for publication by April 2026.

### Ethics and dissemination

As this realist review involves the synthesis of data from existing documents and does not involve the collection of new data from human participants, ethics approval is not required. Findings will be disseminated through open-access, peer-reviewed publications and presentations at national and international conferences. Key stakeholders, including CANO-ACIO, ISNCC and ONS, will be actively engaged to ensure the relevance of the findings and to maximise their impact on nursing practice.

## Discussion

### Novelty of the review

This realist review offers a novel perspective, focusing on CPE interventions aimed at increasing the uptake of EBPs among oncology nurses and seeking to understand how specific implementation strategies may influence this uptake. The review adopts an integrative approach that combines empirical research, grey literature and active engagement from an Expert Advisory Committee comprised of oncology nurses in various roles (eg, nurse navigators, managers, nurse practitioners), decision-makers and researchers in knowledge mobilisation and implementation science. This committee will be consulted at multiple stages to generate actionable insights and improve the practical relevance of the findings for knowledge users.

To ensure methodological transparency and rigour, the review employs a multistage process to assess the relevance, richness and rigour of included documents. In addition, the TAPUPASM framework^73^ will be used to guide the appraisal of rigour, to encourage improved reporting standards in future realist reviews.

## Supplementary material

10.1136/bmjopen-2025-110800 online supplemental file 1

10.1136/bmjopen-2025-110800 online supplemental file 2

10.1136/bmjopen-2025-110800 online supplemental file 3

10.1136/bmjopen-2025-110800 online supplemental file 4

10.1136/bmjopen-2025-110800 online supplemental file 5
