# Supplementary material for: How continuing professional education interventions enhance the uptake of evidence-based practices among oncology nurses: a realist review protocol
Source: BMJ Open. 2026 May 27;16(5):e110800. doi: 10.1136/bmjopen-2025-110800 (PMC13218131; doi:10.1136/bmjopen-2025-110800)
Supplement: online supplemental file 2 [file bmjopen-16-5-s002.docx]

Supplementary Material 2: Initial Programme Theories

| **Table 1. Initial Programme Theories** |  |
| --- | --- |
| TDF Domains (63) - Definitions | Initial Programme Theories (IPT) |
| **Knowledge** - An awareness of the existence of something | 1. **If** nurses are aware of and can relate to concrete examples of EBPs, **then** they will be more likely to apply EBPs in cancer care. (Knowledge of task environment) 2. **If** nurses understand the clinical situations in which a given EBP is most applicable, **then** they will be more likely to apply EBPs in cancer care. (Knowledge of task environment) |
| **Skills** - An ability or proficiency acquired through practice | 1. **If** nurses possess the skills to apply EBPs during routine cancer care, **then** they will be more likely to apply EBPs in cancer care. (Competence) 2. **If** nurses receive training in EBPs, coupled with timely feedback, **then** they will be more likely to apply EBPs in cancer care. (Skills development) |
| **Social/Professional Role and Identity** - A coherent set of behaviours and displayed personal qualities of an individual in a social or work setting | 1. **If** nurses believe the use of EBPs is a key aspect of their professional role, **then** they will be more likely to apply EBPs in cancer care. (Professional identity) 2. **If** nurses believe that being evidence-based practitioners is recognized and supported by other nurses, **then** they will be more likely to apply EBPs in cancer care. (Group identity) |
| **Beliefs about capabilities** - Acceptance of the truth, reality, or validity about an ability, talent, or facility that a person can put to constructive use | 1. **If** nurses believe that they have the necessary knowledge to apply EBPs, **then** they will be more likely to apply EBPs in cancer care. (Perceived competence) 2. **If** nurses feel confident in their skills, **then** they will be more likely to apply EBPs in cancer care. (Self-efficacy) |
| **Optimism** - The confidence that things will happen for the best or that desired goals will be attained | 1. **If** nurses perceive that using EBPs in cancer care will, over time, become more efficient and embedded in their daily workflow, **then** they will be more committed to starting the change now. (Optimism) 2. **If** nurses perceive that EBPs will not realistically become integrated into their workflow due to systemic or contextual barriers, **then** they will be less committed to starting the change now. (Pessimism) |
| **Beliefs about Consequences** - Acceptance of the truth, reality, or validity about outcomes of a behaviour in a given situation | 1. **If** nurses believe that using EBPs is safe, **then** they will be more likely to apply EBPs in cancer care. (Consequences) 2. **If** nurses believe that EBPs enhance patient outcomes, **then** they will be more likely to apply EBPs in cancer care. (Outcomes expectancies) |
| **Reinforcement** - Increasing the probability of a response by arranging a dependent relationship, or contingency, between the response and a given stimulus | 1. **If** nurses are held accountable through rewards for their use of EBPs, **then** they will be more likely to apply EBPs in cancer care. (Incentives) 2. **If** nurses are held accountable through oversight for their use of EBPs, **then** they will be more likely to apply EBPs in cancer care. (Sanctions) 3. **If** peers openly encourage the use of EBPs, **then** nurses will be more likely to apply EBPs in cancer care. (Reinforcement) |
| **Intentions** - A conscious decision to perform a behaviour or a resolve to act in a certain way | 1. **If** nurses form sustained intentions to integrate EBPs into their practice, **then** they will be more likely to apply them in cancer care. (Stability of intentions) 2. **If** nurses’ intentions to use EBPs are unstable and easily influenced by workload or organizational barriers, **then** they will be less likely to apply them in cancer care. (Stability of intentions) |
| **Goals** - Mental representations of outcomes or end states that an individual wants to achieve | 1. **If** nurses perceive EBPs use as a key organizational priority, **then** are more likely to adopt it as a personal goal to apply EBPs in cancer care. (Goal priority) 2. **If** nurses perceive EBPs as conflicting with other professional goals (e.g., efficiency, time management), **then** they will be less likely to apply them in cancer care. (Goal conflict) |
| **Memory, Attention and Decision Processes** - The ability to retain information, focus selectively on aspects of the environment and choose between two or more alternatives | 1. **If** EBPs are embedded into daily workflows and prompts, **then** nurses will be more likely to apply them during cancer care. (Decision making) 2. **If** nurses are overwhelmed by competing demands, **then** they may be less likely to apply EBPs in cancer care. (Cognitive overload) 3. **If** EBPs are presented with clear, actionable options, **then** nurses will be more likely to apply them into cancer care. (Decision making) |
| **Environmental Context and Resources** - Any circumstance of a person's situation or environment that discourages or encourages the development of skills and abilities, independence, social competence, and adaptive behaviour | 1. **If** there are sufficient human resources (e.g., support staff or clinical educators), **then** nurses will be more likely to apply EBPs in cancer care. (Resources) 2. **If** the organizational culture actively promotes EBP use, **then** nurses will be more likely to apply EBPs in cancer care. (Organizational culture) 3. **If** nurses have a manageable workload that allows time for EBP-related activities, **then** they will be more likely to apply EBPs in cancer care. (Environmental stressors) |
| **Social Influences** - Those interpersonal processes that can cause individuals to change their thoughts, feelings, or behaviours | 1. **If** peers in nursing express value for EBP, **then** nurses may feel more accountable and more likely to apply EBPs in cancer care. (Social pressure) 2. **If** champions in nursing on site promote the use of EBPs, **then** nurses will be more likely to apply EBPs in cancer care. (Modelling) 3. **If** nurses perceive strong leadership support for EBP within their organization, **then** they will be more likely to apply EBPs in cancer care. (Power) |
| **Emotion** - A complex reaction pattern, involving experiential, behavioural, and physiological elements, by which the individual attempts to deal with a personally significant matter or event | 1. **If** nurses are apprehensive about change, **then** they will be less likely to apply EBPs in cancer care. (Fear) 2. **If** nurses feel disengaged in their current roles, **then** they may be more motivated to apply EBPs in cancer care as a way to improve their professional environment. (Affect) 3. **If** nurses feel angry or resentful regarding their workload and work conditions, **then** they will be less likely to apply EBPs in cancer care. (Affect) |
| **Behavioural Regulation** - Anything aimed at managing or changing objectively observed or measured actions | 1. **If** nurses receive regular, constructive feedback on their use of EBPs in cancer care, **then** they are more likely to adjust and apply EBPs in cancer care. (Breaking habit) 2. **If** organizations implement systems that actively monitor EBP, **then** nurses may be more likely to apply consistent EBPs in cancer care. (Breaking habit) |
| EBP: Evidence-Based Practices, IPT: Initial Programme Theories, TDF: Theoretical Domains Framework | |
