# Supplementary material for: How continuing professional education interventions enhance the uptake of evidence-based practices among oncology nurses: a realist review protocol
Source: BMJ Open. 2026 May 27;16(5):e110800. doi: 10.1136/bmjopen-2025-110800 (PMC13218131; doi:10.1136/bmjopen-2025-110800)
Supplement: online supplemental file 3 [file bmjopen-16-5-s003.docx]

# Supplementary Material 3: Initial Queries to Expert Advisory Committee

As a reminder, this realist review aims to investigate how, why, for whom, and under what circumstances continuing professional education (CPE)—such as online modules or in-service training—contributes to the uptake of evidence-based practices (EBPs) in oncology.

These practices may include clinical procedures such as dressing changes or the intravenous administration of cancer treatments, professional behaviors like interprofessional communication, or documentation in patient records. To support the integration of EBPs, various strategies may be implemented—for example, clinical audits, reminder systems, cultivating a positive organizational culture, or reinforcing the value of peer support.

Your insights will enhance our understanding of how these complex processes unfold across diverse practice settings.

1. Which **three components are most critical** to making a CPE effective in supporting the uptake of EBPs in oncology?
2. What are the **three most significant challenges** that educators or implementers encounter when using CPE programs to promote the uptake of EBPs in oncology?
3. Are there **specific contextual factors or considerations** that should be addressed to promote the uptake of EBPs through CPE in rural, remote, or non-specialized oncology settings?
4. What **delivery-related factors**—such as format (virtual vs. in-person), timing (e.g., availability outside of work hours), or accessibility (e.g., language, digital literacy)—influence the uptake of EBPs during CPE?

Please let me know if you would rather have a quick chat about your thoughts on these questions instead of responding via email.
