# Supplementary material for: How continuing professional education interventions enhance the uptake of evidence-based practices among oncology nurses: a realist review protocol
Source: BMJ Open. 2026 May 27;16(5):e110800. doi: 10.1136/bmjopen-2025-110800 (PMC13218131; doi:10.1136/bmjopen-2025-110800)
Supplement: online supplemental file 4 [file bmjopen-16-5-s004.docx]

# Supplementary Material 4: Search Strategy in MEDLINE (Ovid)

1     exp Nursing/

2     exp Nurses/

3     exp Nursing Staff/

4     (nurse or nurses or nursing).ti,ab,kf.

5     1 or 2 or 3 or 4

6     exp Neoplasms/

7     (cancer* or oncolog* or neoplas* or carcinoma* or sarcoma* or tumor* or tumour* or lesion* or metastatic* or metastasi* or lymphoma* or leukemia* or leukaemia* or lymphoma* or hodgkin* or myeloma* or melanoma* or mesothelioma* or adenolymphoma* or adenoma* or adenomyoepithelioma* or adenomyoma* or adenosarcoma* or carcinosarcoma* or hepatoblastoma* or mesenchymoma* or myoepithelioma* or blastoma* or thymoma* or angiomyolipoma* or lipoma* or liposarcoma* or chondroblastoma* or chondroma* or chondrosarcoma* or mastocytos* or myxoma* or oseoblastoma* or osteochondroma* or osteoma* or osteosarcoma* or fibroma* or fibrosarcoma or leiomyoma* or leiomyomatos* or leiomyosarcoma* or myoma* or myosarcoma* or lymphangioleiomyomatos* or dermatofibrosarcoma* or hemangiosarcoma* or teratoma* or carcinoid* or neurilemmoma* or paraganglioma* or germinoma* or glioma* or astrocytoma* or glioblastoma* or ependymoma* or ganglioma* or medulloblastoma* or oligodendroglioma* or neuroblastoma* or pinealoma* or retinoblastoma* or craniopharyngioma* or choriocarcinoma* or insulinoma* or cystadenoma* or cystadenocarcinoma* or meningioma* or plasmacytoma* or angiofibroma* or hemangioma* or hemangioendothelioma* or hemangiosarcoma*).ti,ab,kf,jw.

8     6 or 7

9     Education, Continuing/

10    inservice training/ or staff development/

11    ((continuing adj4 (education or development)) or ((inservice or in service) adj3 train*) or ((staff or worker* or employee*) adj3 (education or development)) or (cpd or cpe)).ti,ab,kf.

12    9 or 10 or 11

13    5 and 8 and 12

14    evidence-based practice/ or evidence-based medicine/

15    (((evidence* base* or evidence* informed) adj4 (practice* or healthcare or health care or medicine)) or (ebm or ebp)).ti,ab,kf.

16    14 or 15

17    5 and 8 and 16

18    Oncology Nursing/ or (4 and 7)

19    18 and 12

20    18 and 16

21    Evidence-Based Nursing/

22    (((evidence* base* or evidence* informed) adj4 nurs*) or ebn).ti,ab,kf.

23    21 or 22

24    8 and 23

25    13 or 17 or 19 or 20 or 24
