# Supplementary material for: How continuing professional education interventions enhance the uptake of evidence-based practices among oncology nurses: a realist review protocol
Source: BMJ Open. 2026 May 27;16(5):e110800. doi: 10.1136/bmjopen-2025-110800 (PMC13218131; doi:10.1136/bmjopen-2025-110800)
Supplement: online supplemental file 5 [file bmjopen-16-5-s005.docx]

Supplementary Material 5: Data Extraction Form

1. **DOCUMENT ID**
2. **AUTHORS**

- Names, year published

1. **DOCUMENT TYPE**
   - Methodology papers
   - Empirical papers (i.e. qualitative, quantitative or mixed methods studies)
   - Grey literature (i.e. reports, policies, briefings, conference proceedings)
2. **CONTEXT**

- Country
  - Name of country
    - Must be a high-income or upper-middle-income country, **see World Bank classification (1)**
  - Not reported
- Health system level(s)
  - National
  - Provincial
  - Local
  - Other?
  - Not reported
- Design
  - State the design used
    - Quantitative such as RCT, quasi-experimental, pre-experimental, cross-sectional, longitudinal, cohort.
    - Qualitative: Ethnography, grounded theory, phenomenology, case study,
    - Mixed-methods: Convergent (concurrent), explanatory sequential (Quant → Qual), exploratory sequential (Qual → Quant), embedded, multiphase
  - If unclear = Not reported
- Population
  - Description of population
    - Healthcare professionals
      - Must be relevant to oncology nurses working in oncology
    - Patients
      - Dx
      - Age
  - Sample size for all groups
- Intervention
  - Briefly describe the main intervention
    - Must be related to a CPE intervention
  - Describe the control group/other interventions if relevant
  - If unclear = Not reported
- Implementation strategy
  - Choose at least one implementation strategy from the **ERIC Taxonomy (2)** <https://implementationscience.biomedcentral.com/articles/10.1186/s13012-015-0209-1/tables/3>
  - State all implementation strategies relevant to the implementation, uptake or sustainability of EBPs in the context of CPE interventions

1. **OUTCOMES**

- Implementation outcomes (3) such as:
  - **Acceptability** - Perception among implementation stakeholders that a given treatment, service, practice, or innovation is agreeable, palatable, or satisfactory
  - **Adoption** – Intention, initial decision, or action to try or employ an innovation or evidence-based practice
  - **Appropriateness** - Perceived fit, relevance, or compatibility of the innovation or evidence-based practice for a given practice setting, provider, or consumer; and/or perceived fit of the innovation to address a particular issue or problem
  - **Costs** - Cost impact of an implementation effort
  - **Feasibility** - Extent to which a new treatment, or an innovation, can be successfully used or carried out within a given agency or setting
  - **Fidelity** - Degree to which an intervention was implemented as it was prescribed in the original protocol or as it was intended by the program developers
  - **Penetration** - Integration of a practice within a service setting and its subsystems
  - **Sustainability** - Extent to which a newly implemented treatment is maintained or institutionalized within a service setting’s ongoing, stable operations
- Provider-level outcomes (4) such as:
  - **Knowledge** - An awareness of the existence of something
  - **Skills** - An ability or proficiency acquired through practice
  - **Beliefs about capabilities (self-efficacy)** - Acceptance of the truth, reality, or validity about an ability, talent, or facility that a person can put to constructive use
  - **Beliefs about consequences** - Acceptance of the truth, reality, or validity about outcomes of a behaviour in a given situation
  - **Intentions** - A conscious decision to perform a behaviour or a resolve to act in a certain way
- Patient-level outcomes such as:
  - **Patient-reported outcome measures (PROMs)**
    - Pain
    - Anxiety, depression, stress
    - Activity level
    - Nausea and vomiting
  - **Patient-reported experience measures (PREMs)**
    - Satisfaction
    - Information
    - Involvement in care
    - Communication

**References**

1. World Bank. World Bank Country and Lending Groups 2025 [Available from: <https://datahelpdesk.worldbank.org/knowledgebase/articles/906519-world-bank-country-and-lending-groups>.

2. Powell BJ, Waltz TJ, Chinman MJ, Damschroder LJ, Smith JL, Matthieu MM, et al. A refined compilation of implementation strategies: results from the Expert Recommendations for Implementing Change (ERIC) project. Implementation Science. 2015;10:1-14.

3. Proctor E, Silmere H, Raghavan R, Hovmand P, Aarons G, Bunger A, et al. Outcomes for implementation research: conceptual distinctions, measurement challenges, and research agenda. 2011;38:65-76.

4. Cane J, O’Connor D, Michie S. Validation of the theoretical domains framework for use in behaviour change and implementation research. Implementation Science. 2012;7(1):1-17.
