# Supplementary material for: How continuing professional education interventions enhance the uptake of evidence-based practices among oncology nurses: a realist review protocol
Source: BMJ Open. 2026 May 27;16(5):e110800. doi: 10.1136/bmjopen-2025-110800 (PMC13218131; doi:10.1136/bmjopen-2025-110800)
Supplement: Supplementary file 6 [file bmjopen-2025-110800.reviewer_comments.pdf]

PEER REVIEW HISTORY

BMJ Open publishes all reviews undertaken for accepted manuscripts. Reviewers are asked to complete a checklist review form and are provided with free text boxes to elaborate on their assessment. These free text comments are reproduced below.

ARTICLE DETAILS

Title (Provisional)

How Continuing Professional Education Interventions Enhance the Uptake of Evidence-Based Practices Among Oncology Nurses: A Realist Review Protocol

Authors

Vinette, Billy; Fontaine, Guillaume; Auger, Louis-Pierre; Quaiattini, Andrea; Thomas, Aliki

---

VERSION 1 - REVIEW

Reviewer 1

Name Abraham, Monnie

Affiliation St Jude Children's Research Hospital, Department of Global Pediatric Medicine

Date 15-Dec-2025

COI None

Thank you for the opportunity to review your protocol.

This review aims to explore the mechanisms and contextual factors that influence the success of continuing professional education in supporting the uptake of EBP among oncology nurses. This study is relevant and timely, addressing a critical yet often underexplored dimension of oncology nursing: EBP uptake. The methodology is well aligned with realist review principles.

The protocol is well written and methodologically sound. However, some aspects require further clarification and strengthening regarding the study's aim and methodology.

Major:
The aim of the study:
- The study aim in the abstract is to assess the context and mechanisms that positively and negatively influence the uptake of EBP. In contrast, the aim in the main document is to evaluate only the positive outcomes.

Clarity of methodology:

- The abstract mentions peer-reviewed and grey literature from high-income countries, while the main document also includes upper-middle-income countries.
- There are details on how peer-reviewed articles will be collected, but none on how grey literature will be collected.
- More clarity is needed on how data will be analyzed since the data for "context" and "outcome" will be extracted using the data extraction sheet, while "mechanism" details will be collected through coding in MAXQDA.
- Suggest including "Continuing nursing education" in the literature search strategy

Minor:
- Include the table titles in the main document. E.g., Document screening form on line 347 instead of a standardized form

---

**Reviewer**	**2**
**Name**	**Cooper, Christina**
**Affiliation**	**Northumbria University, SWECW**
**Date**	**13-Mar-2026**
**COI**	**None**

This is a very well written protocol which is easy to follow and addresses an important contemporary issue. I am happy to recommend this protocol for publication.

VERSION 1 - AUTHOR RESPONSE

Comment 4: The study aim in the abstract is to assess the context and mechanisms that positively and negatively influence the uptake of EBP. In contrast, the aim in the main document is to evaluate only the positive outcomes.
Response: We have standardized the aims within the abstract and the primary text to ensure consistency. This revision more clearly demonstrates our intent to examine the factors that both facilitate and hinder the uptake of EBPs.

Comment 5: The abstract mentions peer-reviewed and grey literature from high-income countries, while the main document also includes upper-middle-income countries.
Response: Thank you for this observation. We have removed the mention of upper-middle-income countries to ensure that the included studies share comparable socioeconomic and healthcare environments. This adjustment allows for a more consistent evaluation of how specific contexts and resources interact with the contexts and mechanisms under study.

Comment 6: There are details on how peer-reviewed articles will be collected,

but none on how grey literature will be collected.
Response: We appreciate this feedback. We have explicitly detailed our grey literature strategy in the manuscript (lines 295-297). This includes a systematic screening of the first 250 results from Google Scholar and a targeted manual search of key oncology nursing repositories (e.g., ONS, ISNCC, CANO, CNSA) to identify relevant reports and unpublished findings. We also added a list of potential websites that will be consulted for reports and unpublished results. “We will also manually search the repositories of major oncology nursing organizations (e.g., Oncology Nursing Society, International Society of Nurses in Cancer Care, Canadian Association of Nurses in Oncology, Cancer Nurses Society of Australia) to retrieve relevant gray literature, reports, and unpublished studies that meet our inclusion criteria.”

Comment 7: More clarity is needed on how data will be analyzed since the data for “context” and “outcome” will be extracted using the data extraction sheet, while “mechanism” details will be collected through coding in MAXQDA.
Response: We have refined the Data Analysis section to provide a clearer roadmap of our analytical process. This revision explicitly details the integration of context, outcome, and mechanism data, clarifying the specific sequence in which these elements are synthesized to develop our CMOCs. The analysis will follow an iterative, two-stage process of theoretical refinement. First, the data extracted via Covidence regarding contexts and outcomes will be used to test the viability of the IPTs. This step involves assessing the coherence of the IPTs against the evidence, identifying recurring patterns, and highlighting gaps or inconsistencies where the data does not align with the initial theories. Once the IPTs are stabilized, a second phase of in-depth coding will be conducted in MAXQDA to identify the latent mechanisms. BV will organize the extracted data into evidence tables, which will then be analyzed Tthrough a retroductive approach combining inductive and deductive reasoning, we will triangulate the coded mechanisms with the previously extracted contexts and outcomes. This synthesis will transform the IPTs into refined CMOCs, articulated as: “if [context]... then [outcome]... because [mechanism]” propositions.

Comment 8: Suggest including “Continuing nursing education” in the literature search strategy
Response: We agree with the reviewer. 'Continuing nursing education' has been added to our search strategy across all databases.

Comment 9: Include the table titles in the main document. E.g., Document screening form on line 347 instead of a standardized form
Response: Table titles have been added throughout the manuscript (e.g., 'Document Screening Form' on line 347) to improve clarity as suggested.
